# *Parascolymia* (Scleractinia: Lobophylliidae) in the Central Paratethys Sea (Vienna Basin, Austria) and its possible biogeographic implications

**DOI:** 10.1371/journal.pone.0132243

**Published:** 2015-07-22

**Authors:** Markus Reuter, Thomas Wiedl, Werner E. Piller

**Affiliations:** Institute for Earth Sciences, University of Graz, Graz, Austria; University of California, UNITED STATES

## Abstract

Palaeobiogeographical and palaeodiversity patterns of scleractinian reef corals are generally biased due to uncertain taxonomy and a loss of taxonomic characters through dissolution and recrystallization of the skeletal aragonite in shallow marine limestones. Herein, we describe a fossil lobophylliid coral in mouldic preservation from the early middle Miocene Leitha Limestone of the Central Paratethys Sea (Vienna Basin, Austria). By using grey-scale image inversion and silicone rubber casts for the visualization of the original skeletal anatomy and the detection of distinct micromorphological characters (i.e. shape of septal teeth, granulation of septocostae) *Parascolymia bracherti* has been identified as a new species in spite of the dissolved skeleton. In the recent era, *Parascolymia* like all Lobophylliidae is restricted to the Indo-Pacific region, where it is represented by a single species. The new species proves the genus also in the Miocene Mediterranean reef coral province. A review of the spatio-temporal relationships of fossil corals related to *Parascolymia* indicates that the genus was probably rooted in the Eastern Atlantic‒Western Tethys region during the Paleocene to Eocene and reached the Indo-Pacific region not before the Oligocene. The revealed palaeobiogeographical pattern shows an obvious congruence with that of *Acropora* and tridacnine bivalves reflecting a gradual equatorwards retreat of the marine biodiversity center parallel to the Cenozoic climate deterioration.

## Introduction

Traditional classification and phylogeny of the Scleractinia Bourne, 1900 depend almost exclusively on macromorphological characters related to the corallite architecture and the integration of corallites within colonies, which can be easily measured on recent and fossil coral skeletons [1, 2]. For the traditional scleractinian families Faviidae Gregory, 1900 and Mussidae Ortmann, 1890, the combined use of morphological and molecular data has, however, revealed extensive homoplasy in almost all macromorphological characters while it turned out that micromorphological characters (e.g. shapes of teeth and granules along the margins and faces of septa) distinguish molecular clades at the family- and subfamily level [2–5]. This result suggests new hypotheses for relationships among genera and families that are unlike those proposed on the basis of conventional taxonomy and is transforming our view on scleractinian biogeography and evolution [6]. For the “mussid” genus *Scolymia* Haime, 1852, once thought to be cosmopolitan [7], it has been shown that Atlantic *Scolymia* are more closely related to the Atlantic “faviid” *Favia* Milne Edwards, 1857 than to Indo-Pacific *Scolymia* [3, 5]. Accordingly, the previous Atlantic and Indo-Pacific genus *Scolymia* [7] has been recently split into three genera distinguished on the basis of the shapes of septal teeth and corresponding microstructure following the molecular phylogeny [5]: (1) *Scolymia* (assigned to the family Mussidae, Atlantic); (2) *Parascolymia* Wells, 1964 (assigned to the family Lobophylliidae Dai & Horng, 2009, Indo-Pacific); and (3) *Homophyllia* Brüggemann, 1877 (assigned to the family Lobophylliidae, Indo-Pacific). Previous generic assignments of fossil taxa need to be also re-examined including micromorphological features to resolve spatio-temporal patterns of coral diversity and distribution. Problematically, most fossil corals derived from shallow marine limestone facies do not show distinctive micromorphological characters to be preserved because their primary aragonitic skeletons became transformed into secondary calcite and fully cemented. Therefore the timing of the separation between the Atlantic and Pacific clades is difficult to validate [3].

As a prominent feature of modern global biogeography, species diversity of many marine biota reaches a maximum in the Indo-Australian Archipelago (Malaysia, Indonesia, New Guinea, and the Philipines) of the Indo-West Pacific region. This great diversity of marine life reflects the range of shallow marine habitats which include coral reefs along with seagrass meadows and mangroves [8,9]. At present, these most diverse and productive marine ecosystems are under increasing threat from a range of natural and man-made disturbances. Environmental changes are a pervasive part of earth history and the documentation of ancient biogeographic dynamics related to these changes is essential for determining the response of shallow marine ecosystems to current global change as a critical research priority for both Life and Earth Scientists. The molecular and fossil evidence suggest that the majority of extant species in the Indo-Australian Archipelago originated in the Miocene and contradicts the notion of Pleistocene origins of the modern fauna and flora [10]. It is, however, not known precisely when and why this diversity originated [11].

Herein, we report on two fossil *Parascolymia* species of early middle Miocene age from the Central Paratethys Sea (Vienna Basin, Austria). These records of *Parascolymia* from outside the Indo-Pacific region and from a time interval of important biogeographic change in the marine biosphere [10,12–15] are adding more detail for understanding the diversification of the largest modern marine biogeographic province–the Indo-West Pacific.

### Depositional Environment and Palaeoecology

**Fig 1:**
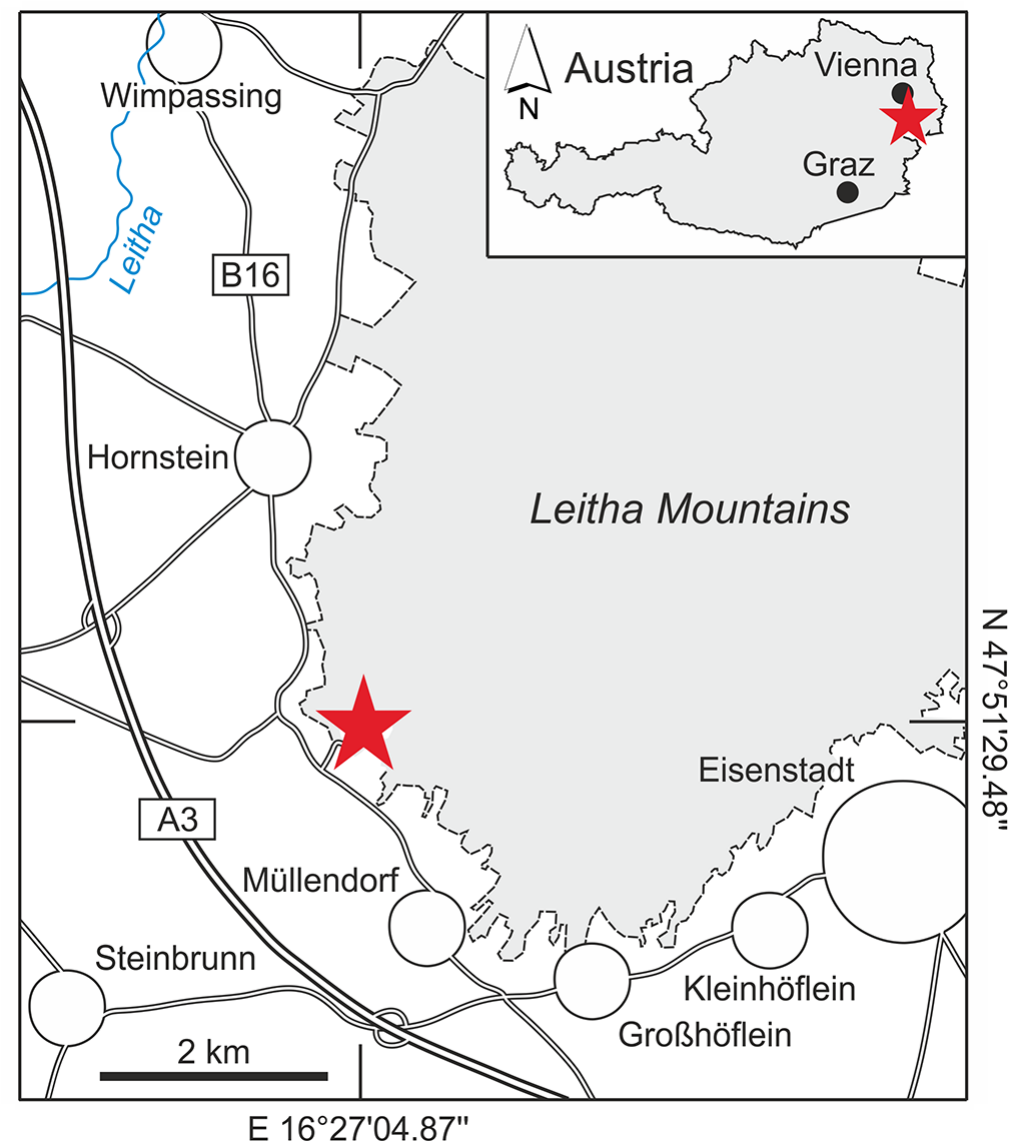
Location of the Müllendorf quarry (asterisk) at the southwestern margin of the Leitha Mountains (Austria).

The Paratethys Sea originated during the latest Eocene and early Oligocene as a northern satellite basin of the Tethys Ocean and spread from the Rhone Basin in France towards Inner Asia during its maximum extent [16]. Facilitated by the warm climate and sea level highstand, tropical coral reef ecosystems extended northwards into the Central Paratethys Sea during the Middle Miocene Climate Optimum (ca. 17‒14 Ma) for the only time in the Neogene [14, 17, 18]. At this time the Trans-Tethyan Trench Corridor connected the Central Paratethys with the Western Tethys/Proto-Mediterranean Sea via Slovenia [19]. Furthermore, an open connection to the Eastern Paratethys and from there into the Western Tethys/Proto-Mediterranean Sea and Eastern Tethys/Proto-Indo-Pacific is postulated [16], but this eastern seaway is still controversial [19]. The marginal reef coral communities of the Central Paratethys are generally of low diversity (usually less than 5 genera at the same site) and characterized by a low framework-building capacity. Non-framework forming coral communities and coral carpets dominated while higher diversity (up to 10 coral genera at the same site) coral patch reefs formed just briefly during the climax of the Middle Miocene Climate Optimum along the western coast (Styrian, Slovenian, Vienna basins) and spatially restricted in areas sheltered from siliciclastic input [14, 18, 20–24]. *Parascolymia bracherti* sp. nov. was found in the Müllendorf quarry (N 47°51’29.48”, E 16°27’04.87”) at the southwestern margin of the Leitha Mountains in the Vienna Basin (Austria; Fig 1). This outcrop preserves a record of corallinacean-dominated shallow water carbonates (Leitha Limestone) with intermittent coral-, mollusc- (i.e. *Isognomon*, *Hyotissa*), bryozoan- and siliciclastic-rich intervals [18]. It was deposited on an isolated carbonate platform that developed during the Badenian (Langhian‒early Serravallian) in the area of the present-day Leitha Mountains. The coral derived from coral-corallinacean rudstones with a low content of fine quartz sand [18]. This facies is interpreted to represent a shallow subtidal carbonate sand flat slightly influenced by terrigenous supply, and inhabited by a sparse non-framework forming coral community composed of *Tarbellastraea* and *Acanthastrea* with massive growth forms as well as thin-branching *Porites* [18].

## Material and Methods

The fossil coral was legally collected from a commercial limestone quarry with permission granted by the quarry operator (Mühlendorfer Kreidefabrik Margit Hoffmann-Ostenhof KG). Typical for the limestones at Müllendorf locality is a soft, chalky appearance due to diagenetic leaching and incomplete cementation [25]. In order to stabilize the fragile fossil, the sediment surface was impregnated with a Mowilith solution. Grey-scale image inversion (realized with CorelDRAW X6) was used to visualize original macromorphological features of the dissolved coral skeleton. Further, casts from the fossil were produced using two-component silicone rubber (Essil 2, AXSON GmbH). This sampling technique has been previously successfully applied to molluscs [26–28] and allows restoring micromorphological characters, which are crucial for reliable identification of lobophylliid corals. The fossil (one fragmentary mould in original sediment) and 14 corresponding silicone rubber casts are permanently accessible in the collection of the Geological-Paleontological Department of the Natural History Museum Vienna and stored under the inventory number NHMW 2014/025/0001. The systematic hierarchy follows the revised classification of the reef coral family Mussidae [5]. This systematic account, based on integrated molecular, macro- and micromorphological data, subdivides the previous family Mussidae into Atlantic (Mussidae) vs. Indo-Pacific (Lobophylliidae) groups.

### Nomenclatural Acts

The electronic edition of this article conforms to the requirements of the amended International Code of Zoological Nomenclature, and hence the new names contained herein are available under that Code from the electronic edition of this article. This published work and the nomenclatural acts it contains have been registered in ZooBank, the online registration system for the ICZN. The ZooBank LSIDs (Life Science Identifiers) can be resolved and the associated information viewed through any standard web browser by appending the LSID to the prefix "http://zoobank.org/". The LSID for this publication is: urn:lsid:zoobank.org:pub: C664CFB4-CB78-4496-8EBA-5B2510BD8D14. The electronic edition of this work was published in a journal with an ISSN, and has been archived and is available from the following digital repositories: PubMed Central, LOCKSS.

### Systematic Palaeontology

Class Anthozoa Ehrenberg, 1834

Subclass Hexacorallia Haeckel, 1896

Order Scleractinia Bourne, 1900

Family Lobophylliidae Dai & Horng, 2009

Genus *Parascolymia* Wells, 1964

#### Type species

*Parascolymia vitiensis* (Brüggemann, 1877)

#### Diagnosis

Solitary “mussids” with large calice (>4 cm) and wholly dentate septal margins. Approximately equal dentations (4‒6 per cm on larger septa) and numerous lateral septal granules. Corallite centers with lamellar linkage [29]. Predominantly parathecal, partially septothecal corallite wall; epitheca is absent [5].

#### Differential diagnosis

In the original description of the genus [29], *Parascolymia* differs from *Scolymia* by asexually produced corallites with lamellar rather than trabecular linkage and more numerous and thicker septal granules. Later, lamellar linkage was also observed in *Scolymia* and both genera have been distinguished on the shape of septal teeth and granulation pattern [5]. *Parascolymia* has irregular lobate teeth (elliptical-parallel bases) and rounded, evenly scattered septal granules while *Scolymia* has regular, spine-shaped teeth (circular bases) and spiky, aligned septal granules. *Homophyllia* differs from *Parascolymia* by the narrowly spaced septal teeth (10‒12 per cm on larger septa) and small calices (<4 cm) [5,29]. The solitary lobophylliid *Cynaria* is also similar to *Parascolymia*, but adult specimens exhibit a very high calice relief (>10 mm; *Parascolymia* 4‒10 mm) associated with well-developed septal lobes [30]. The exclusively fossil genus *Indophyllia* Gerth, 1921 [5], previously referred to the family Mussidae [7], is disk-shaped and flat in the young stage, but in the adult stage the coral is growing cylindrically upward, while forming an epitheca on its wall [31]. The presence of an epitheca distinguishes also the extinct mussiid genus *Syzygophyllia* Reuss, 1860 and the genus *Antillia* Duncan, 1864 (Scleractinia insertae sedis [32]) from *Parascolymia*. *Antillia* further exhibits regular large rounded septal teeth in contrast to *Parascolymia*. The fossil solitary mussiid *Leptomussa* d’Archiardi, 1867 developed only scarcely an epitheca but a columella is absent [33,34].

*Parascolymia bracherti* Reuter sp. nov.

urn:lsid:zoobank.org:act: 09EDF995-822C-43B0-932A-7E57CC5EDF92

Figs 2–5

**Fig 2:**
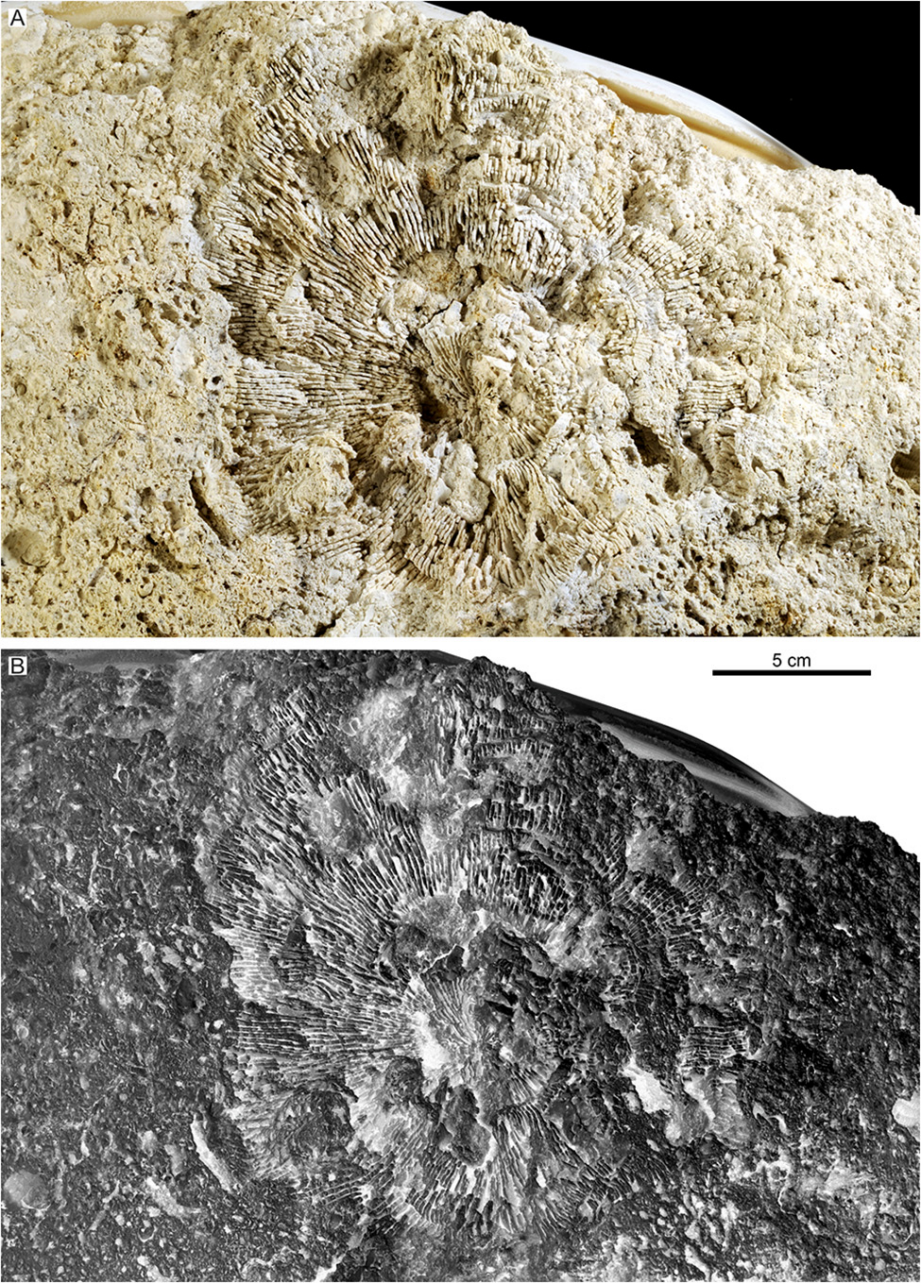
*Parascolymia bracherti* sp. nov., holotype (NHMW 2014/0205/0001). (A) overview of the corallite mould; (B) inverted grey-scale image of (A) giving a three-dimensional impression of the original corallite structure.

**Fig 3:**
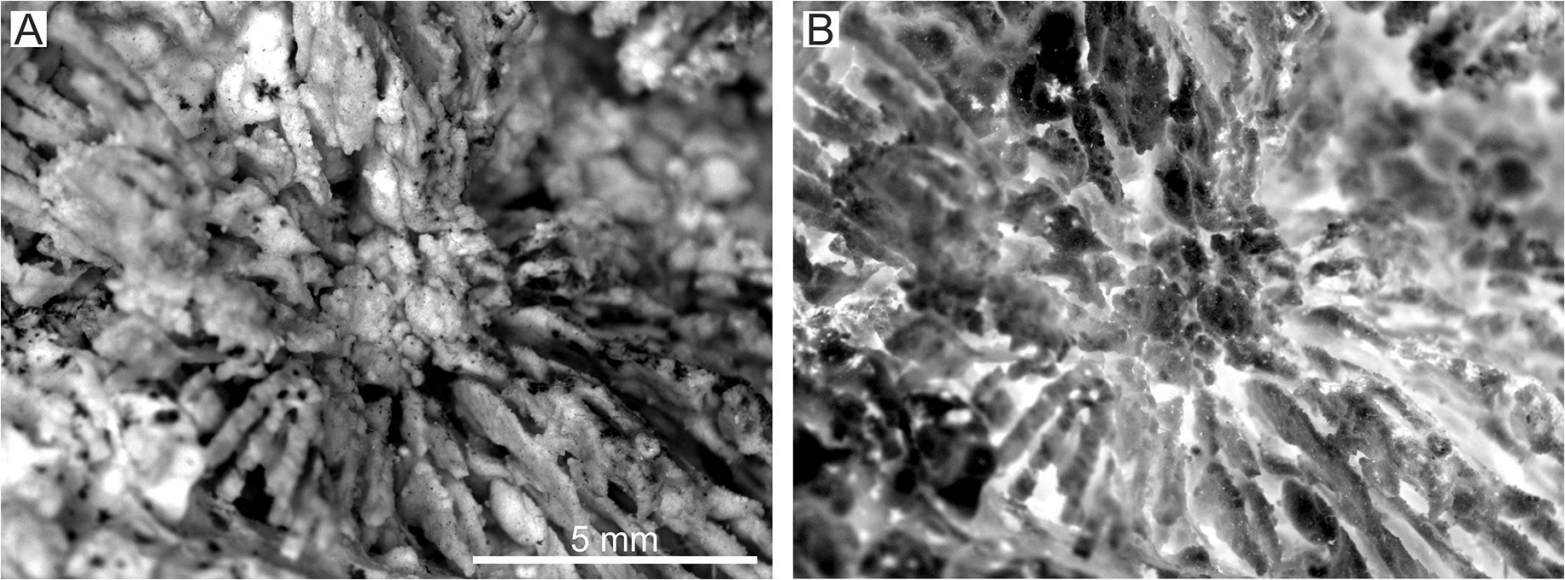
*Parascolymia bracherti* sp. nov., columella. (A) detail view of the corallite center (mould). The irregular knobby structure likely represents sediment-sealed interstices between former lamellar linkages; (B) same as (A), but inverted grey-scales to create a positive image of the spongy columella.

**Fig 4:**
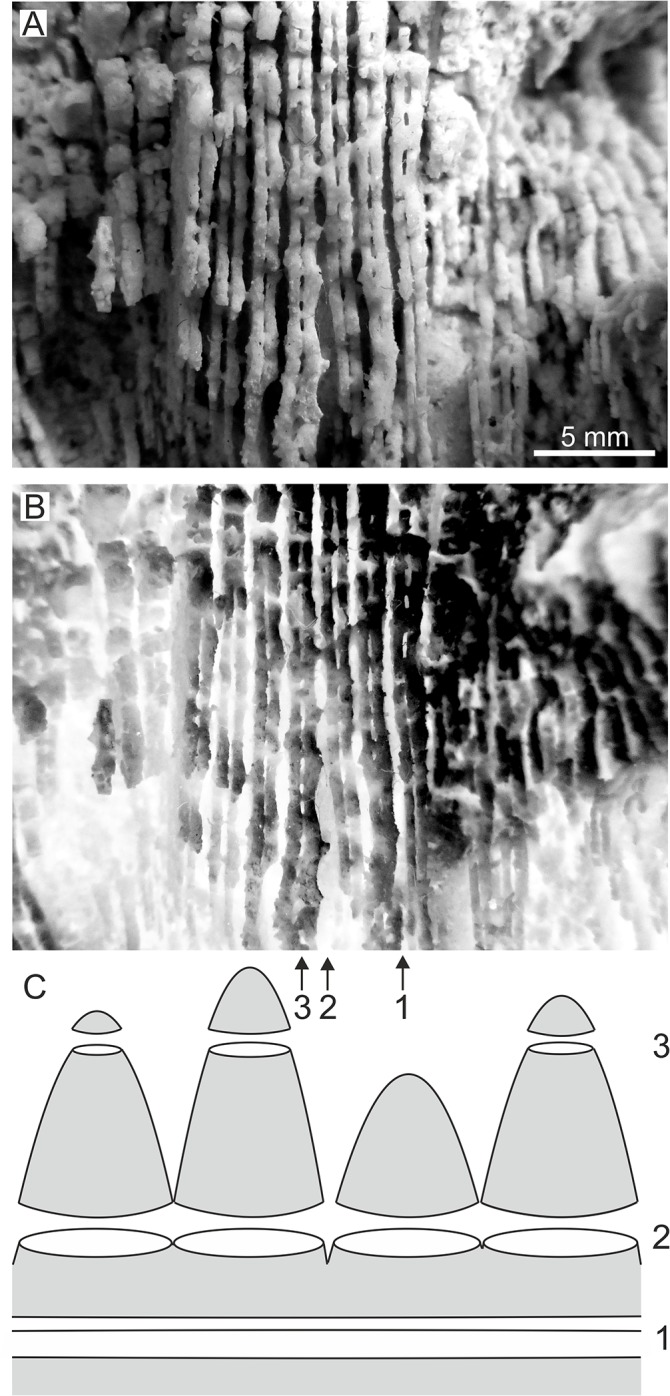
*Parascolymia bracherti* sp. nov., septal dentation. (A) detail view of septa moulds; (B) grey-scale inversion image of (A) showing different outlines of lower and higher order septa; (C) schematic diagram illustrating the relationship between septum profile and septum height (1 = linear profile at the base of a septum, 2 = chain of ovals at the base of septal teeth, 3 = irregularly interrupted pattern at the tip of septal teeth).

**Fig 5:**
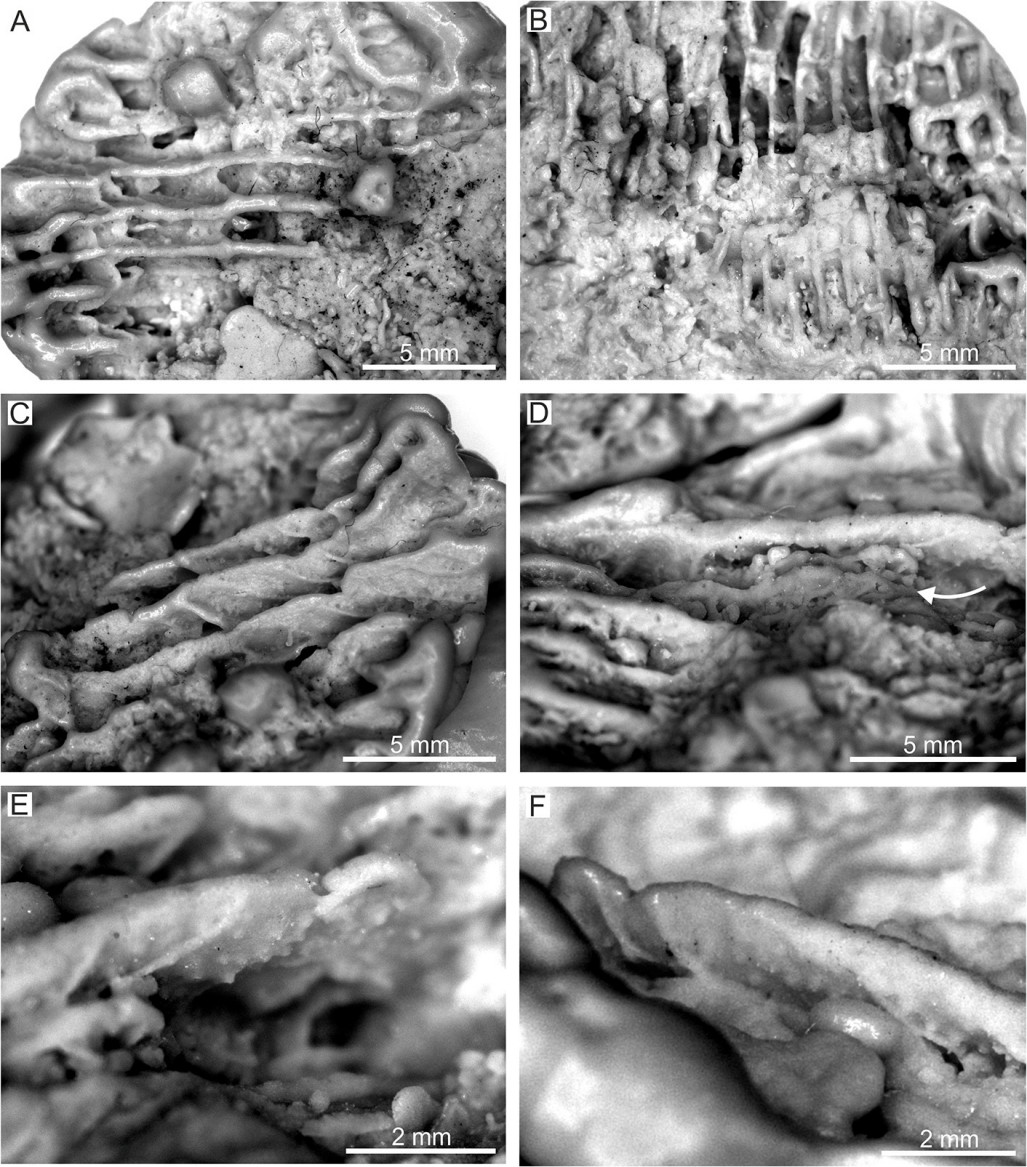
*Parascolymia bracherti* sp. nov., silicone casts. (A, B) parathecal wall; (C, D) blunt saw-like ornamentation of costae margins (arrow in D indicates a costa margin); (E, F) rounded granules on septocostae flanks.

#### Etymology

In honor of the German geologist Thomas C. Brachert in recognition of his research on Miocene shallow marine carbonates in the Mediterranean region.

#### Holotype

NHMW 2014/0205/0001. Fragmentary mould in original sediment and 14 corresponding silicone rubber casts.

#### Horizon and type locality

The specimen comes from the Leitha Limestone in the Müllendorf quarry at the southwestern margin of the Leitha Mountains (Vienna Basin, Austria); section B, bed 5 [18].

#### Diagnosis

Large, monocentric mussid-like corallum with circular outline, parathecal wall and spongy columella. Lower order septocostae with well-developed, wide-spaced irregular lobate septal teeth and rounded granules evenly scattered on the flanks.

#### Description

Mould of a circular, monocentric corallum (30 mm in length, 170×170 mm in diameter) with radial lamellar structure (Fig 2). The lamellar structure represents interstices between former (now dissolved) vertical skeletal elements (septocostae), which are filled by sediment. In the following, we only refer to the mould when we quote the skeletal elements. The straight septocostae radiate towards a small (4×5 mm) spongy columella with indistinctly preserved lamellar linkage (Figs 2 and 3). Cross-sections through nearby septo-costal units, which are cut in the same level, show different profiles due to alternating septa of higher and lower order (Fig 4A and 4B). Lower order septa are thicker and exhibit a relatively regular chain of ovals in cross-section (Fig 4A and 4B) due to the presence of wide-spaced septal teeth (4 per cm), which are cut at their elliptical bases (Fig 4C). In contrast, the profile of higher order septa is irregularly interrupted (Fig 4A and 4B) due to cross-sections through irregular spacing and/or differently high tips of teeth (6‒8 per cm; Fig 4C). This pattern indicates a lower elevation and finer dentation of higher order septa compared to septa of lower order. Already 4 mm above the base of lower order septa from the outer part of the corallum, the imprints of septal teeth indicate a low calice margin. There is no clear corallite wall (Fig 2). However, silicone rubber casts from the bottom of the mould reveal lateral connections between the septa indicating a parathecal wall (Fig 5A and 5B). They further document a blunt saw-like ornamentation of the costae margins (Fig 5C and 5D) and rounded granules evenly scattered on the septocostae flanks (Fig 5E and 5F). Irregular concentric grooves at the base of the fossil represent growth lines on the aboral side.

#### Remarks

In the recent, the genus *Parascolymia* is only represented by *P*. *vitiensis*, which is found in most reef environments throughout the western Indian Ocean from the Maldives to Madagascar and throughout the Pacific from the Indonesian Archipelago to Eastern Australia, French Polynesia and Taiwan, but it is usually uncommon [7,35,36]. The corallum is generally turbinate, but shows a wide variation ranging from subdiscoidal or patellate forms to thick cylindrical columns [29,37]. In subtropical localities it is usually solitary and flat with the calice forming a shallow, conical depression but less than 60 mm in diameter. In the tropics it is larger (single calice specimens up to 100 mm in diameter) and may become polycentric and colonial with a diameter of more than 14 cm [7,35,36]. Septa are usually in five or six orders [30] and become thinner and smaller with finer dentations as the order increases. Lower order septa are strongly and irregularly dentate. Columella is large and spongy [35].

*P*. *vitiensis* has been verified in the Indo-West Pacific region since the early Miocene but many fossil records have been originally described as *Lithophyllia* species (*L*. *grandissima* Felix, 1915; *L*. *margariticola* Klunzinger, 1879; *L*. *sumatrensis* Umbgrove, 1926) [29,38]. Accordingly, Chaix & Cahuzac [39] supposed in their description of *L*. *detrita* that the genus *Lithophyllia* (invalid [38]) is a synonym of *Scolymia*. The only reasons why they did not place *Lithophyllia* in synonymy with *Scolymia* is that the genus *Scolymia* was not precisely defined and has been used in a different manner when *Lithophyllia* was established. Given these complications it cannot ruled out that fossil *Scolymia* and *Lithophyllia* occurrences in the Eastern Atlantic‒Mediterranean area actually relate to *Parascolymia*. For *Scolymia*, this cannot be verified as it is only reported in fossil lists [14,39].

**Fig 6:**
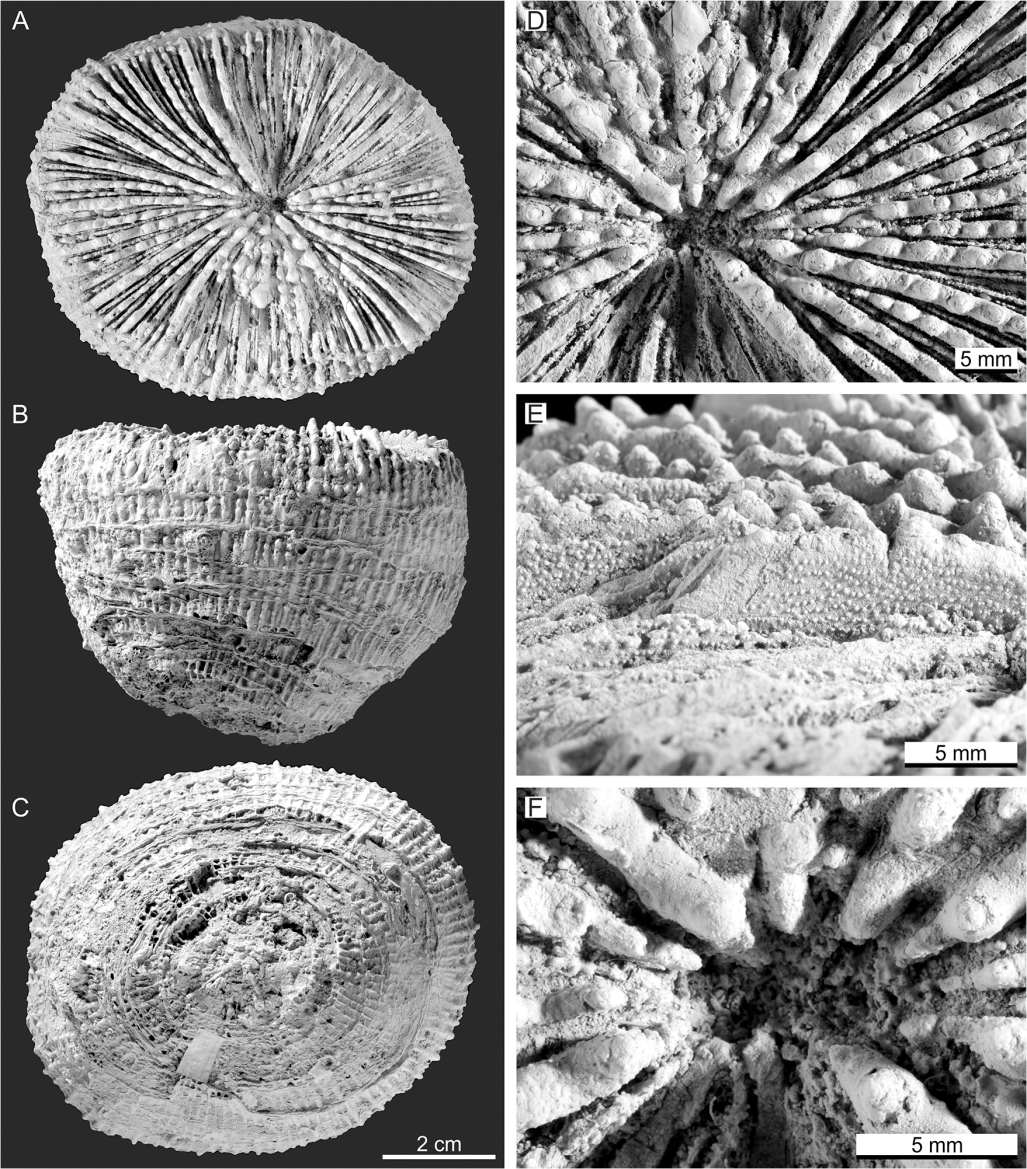
*Lithophyllia ampla*, holotype (NHMW 1872/0013/0036). (A) oral surface; (B) lateral view; (C) aboral surface; (D) top view of septa showing elliptical outlines of the septal teeth; (E) lateral view of the septa showing the triangular shape of septal teeth and rounded granules; (F) spongy columella with lamellar linkage.

*L*. *ampla* Reuss, 1871 occurred contemporaneously with *P*. *bracherti* sp. nov. in the Central Paratethys Sea [40–43]. It has been indicated as a synonym of *Acanthophyllia* Wells, 1937 [34,42], which is synonymous with *Cynarina* according to the WoRMS database [44]. This species is similar in appearance to *P*. *bracherti* sp. nov. The 55 mm high turbinate corallum of the holotype (Fig 6) has a broad elliptical (77×67 mm), shallow calice (Fig 6A) and a small spongy columella (Fig 6A and 6F). Septocostae are arranged in 5‒6 cycles (Fig 6A), have irregular lobate teeth (3‒4 per cm, Fig 6D and 6E) and rounded granules evenly scattered on the margins (Fig 6E). The low calice relief as well as the shape of septal teeth and type of septal granulation indicates rather a *Parascolymia* than a *Cynarina* or *Scolymia* species. We therefore place *Lithophyllia ampla* in synonymy with *Parascolymia*.

*Lithophyllia detrita* (Michelin, 1842) is documented in the Eastern Atlantic‒Western Mediterranean region from the middle Eocene to middle Miocene [39,45,46]. It was recombined from *Anthophyllum detritum* Michelin, 1842 [47] and is characterized by the virtual lack of any wall, a spongy columella, five incomplete septal cycles and a great calice [39]. Wide-spaced septal teeth with an elliptical outline and a low calice relief like *Parascolymia* are also clearly visible in the pictured specimen [39]. Despite this high conformity, *L*. *detrita* cannot be certainly assigned to *Parascolymia* since the nature of the corallite wall is unclear.

*L*. *michelottii* Michelin 1841, *L*. *patula* Sismonda, 1871, and *L*. *gigas* Chevalier, 1961 are other fossil *Lithophyllia* species described from the Miocene of the Mediterranean region [48]. Images of the neotype of *L*. *michelottii* and the holotype of *L*. *gigas* [48] show elliptical-parallel bases of wide-spaced septal teeth; the holotype of *L*. *patula* is lost [48]. However, these three species are described as having partially epithecal walls unlike *Parascolymia* [48].

In contrast, *Lithophyllia robusta* Kühn, 1933 from the Burdigalian of Iran possesses a parathecal wall and has no epitheca, but the calice surface is not preserved [49] and the septa morphology is beyond recognition due to bioerosion and recrystallization (own inspection of the holotype deposited in the Natural History Museum Vienna). Likewise, the septal dentation of *Lithophyllia spinosa* Gerth, 1933 from the Miocene of the Central Indo-Pacific is not preserved. This species is further characterized by a long cylindrical corallum [50] untypical for *Parascolymia*.

It has been further indicated that the fossil Mussiidae genus *Circophyllia* Milne Edwards & Haime, 1848 is a subgenus of *Parascolymia* by renaming *Circophyllia farquharsoni* Latham, 1929 as *Parascolymia (Circophyllia) farquharsoni* [51] (stated without evidence). *Circophyllia* is characterized by a simple subturbinate corallum [52]. The calice is circular and shallow [33]. Septa are broad, numerous, exsert with their calicular edge divided in small obtuse lobes. The columella is large, trabecular below and papillose on surface [33,53]. The corallite wall is septothecal [34] and endothecal dissepiments are numerous [33,53]. *Circophyllia* is distinguished from *Syzygophyllia* by the absence of an epitheca [40]. Costae are thin, nearly equal, simple, and delicately granulated [52,53]. *Circophyllia truncata* Goldfuss, 1826 (type species of the genus *Circophyllia*) specimens in the collection of the Natural History Museum Vienna show a rounded shape of septal granules. The turbinate growth form, the shallow calice relief, irregular lobate septal teeth, and rounded granules as well as the absence of an epitheca are common features of *Circophyllia* and *Parascolymia*. However, the *Circophyllia* wall has been characterized by septal thickening (septothecal) [34], while that of *Parascolymia* is mostly composed of dissepiments (parathecal) [5]. But, numerous endothecal dissepiments are also present in *Circophyllia* [33,53], and *Parascolymia* walls are also partially septothecal [5]. Dissimilar to *Parascolymia vitiensis* and *Parascolymia ampla* (Reuss, 1871) comb. nov. the columella surface of *Circophyllia* is papillose.

*P*. (*Circophyllia) farquharsoni*, which has been originally described from the Oligocene of Somalia [54], has a large, flat corallum, with its diameter (67×75 mm) four times its height (17 mm). Septa very numerous, belonging to nine cycles and numbering about 192; only septa of the first cycle reach the columella, but the first three cycles are of almost equal thickness. The remaining septa are thin and dentate. Columella large and oval (4×7 mm), composed of wavy trabeculae. However, the original description provides neither information about the wall structure nor the morphology of the septal teeth and granules because the type specimen has undergone much secondary calcification, the lobes on the free surfaces of the septa have been worn flat due to weathering and the interstices have been filled up with sediment [54]. All other *Circophyllia* species were originally described from the Eocene of Europe and largely restricted to the Eastern Atlantic and Western Tethys areas (Table 1). From the Eastern Tethys region only two Eocene *Circophyllia* occurrences are mentioned, one from Socotra Island (Gulf of Aden) [55] and the other from Papua New Guinea [56]. The record from Socotra Island is from a fossil list, which is based on an unpublished geological mapping report and not reproducible since all rock samples and specimens collected have been dispersed [55] and the fossils are not depicted. Likewise, the outlier from Papua New Guinea should be treated with caution since its age and taxonomic features are not well constrained (a complete and a partial cross-section of one specimen in a stratigraphically isolated pebble from the bed of Fly River, not figured). A single *Circophyllia d’archiardii* Oppenheim, 1901 is further mentioned from Somalia [54] representing the only Oligocene record of this species. However, this coral is neither appropriately described *(“This species is represented by a single coral which seems to be identical with the descriptions and figures of C*. *d’archiardii*.*”*) nor pictured.

**Table 1:** Fossil record of *Parascolymia* and possibly related *Scolymia*, *Circophyllia* and *Lithophyllia* in the Eastern Atlantic‒Mediterranean and Indo-West Pacific regions. Due to a number of obscure species designations and synonyms the list considers only *Circophyllia* species in the Palaeobiology Database [65].

taxa	synonym	stratigraphic range	localities	geographic region	sources
*Parascolymia vitiensis* (Brüggemann, 1877)	*Protolobophyllia japonica* (Yabe & Sugiyama, 1931)	Pliocene	Japan (Ryukyu Islands)	Western Pacific	[29]
*Parascolymia vitiensis* (Brüggemann, 1877)		Pliocene	Papua New Guinea	Central Indo-Pacific	[75]
*Parascolymia vitiensis* (Brüggemann, 1877)	*Lithophyllia grandissima* Felix, 1915	Pliocene	Borneo	Central Indo-Pacific	[76]
*Parascolymia vitiensis* (Brüggemann, 1877)	*Lithophyllia grandissima* Felix, 1915	early Pliocene	Java	Central Indo-Pacific	[77]
*Lithophyllia spinosa* ^?^ Gerth, 1921		Miocene	Java	Central Indo-Pacific	[50]
*Parascolymia vitiensis* (Brüggemann, 1877)	*Lithophyllia sumatrensis* Umbgrove, 1926	Miocene	Sumatra	Central Indo-Pacific	[78]
*Parascolymia vitiensis* (Brüggemann, 1877)	*Lithophyllia grandissima* Felix, 1915	late Miocene	Java	Central Indo-Pacific	[79]
*Parascolymia* cf. *ampla* (Reuss, 1871)	*Lithophyllia cf*. *ampla* Reuss, 1871	middle Miocene	Turkey (Cilicia)	Eastern Mediterranean Sea	[57]
*Lithophyllia detrita* (Michelin, 1842)		Serravallian	SW France (Aquitaine Basin)	Eastern Atlantic	[46]
*Parascolymia bracherti* Reuter sp. nov.		Badenian (late Langhian‒early Serravallian)	Austria (Vienna Basin)	Central Paratethys	this study
*Parascolymia ampla* (Reuss, 1871)	*Lithophyllia ampla* Reuss, 1871	Badenian (Langhian)	Austria (Styrian Basin)	Central Paratethys	[41]
*Parascolymia ampla* (Reuss, 1871)	*Lithophyllia ampla* Reuss, 1871, *Acanthophyllia ampla* (Reuss, 1871)	Baden (Langhian‒early Serravallian)	Romania	Central Paratethys	[40,43]
cf. *Parascolymia ampla* (Reuss, 1871)	cf. *Acanthophyllia ampla* (Reuss, 1871)	Badenian (Langhian‒early Serravallian)	Hungary	Central Paratethys	[42]
*Lithophyllia spinosa* ^?^ Gerth, 1921		early Miocene	Java	Central Indo-Pacific	[79]
*Lithophyllia spinosa* ^?^ Gerth, 1921		Burdigalian	Java	Central Indo-Pacific	[79]
*Lithophyllia robusta* ^?^ Kühn, 1933		Burdigalian	Iran	Tethyan Seaway	[49]
*Parascolymia vitiensis* (Brüggemann, 1877), *P*. *(Circophyllia) farquharsoni* (Latham, 1921)		Burdigalian	Iran (Makran)	Western Indian Ocean	[51]
*Parascolymia (Circophyllia) farquharsoni* (Latham, 1921), *Circophyllia d’archiardii* ^?^ Oppenheim, 1901	*Circophyllia farquharsoni* Latham, 1921	Oligocene	Somalia	Eastern Tethys	[54]
*Lithophyllia detrita* (Michelin, 1842), *Scolymia* aff. *lacera lacera* (Pallas, 1766), *Scolymia* nov. sp. 1		Chattian	SW France (Aquitaine Basin)	Eastern Atlantic	[39,46]
*Circophyllia* sp.^?^		Eocene	Papua New Guinea	Southeast Asia	[56]
*Circophyllia d’archiardii* ^?^ Oppenheim, 1901		Eocene	Gulf of Aden (Socotra Island)	Eastern Tethys	[55]
*Circophyllia* cf. *costata* Alloiteau, 1949, C. *d’archiardii* Oppenheim, 1901, *C*. cf. *gibba* Oppenheim, 1901, *C*. *hantkeni* Reuss, 1870		late Eocene	Hungary	Western Tethys	[80]
*Circophyllia d’archiardii* Oppenheim, 1901		late Eocene	Croatia	Western Tethys	[81]
*Lithophyllia detrita* (Michelin, 1842)		middle Eocene	Spain (South Pyrenean Foreland Basin)	Western Tethys	[45]
*Circophyllia truncata* Goldfuss, 1826		Lutetian‒Bartonian	France (Atlantic Coast)	Eastern Atlantic	[82]
*Circophyllia d’archiardii* Oppenheim, 1901		Lutetian	Italy	Western Tethys	[83]
*Circophyllia truncata* Goldfuss, 1826	*Ghirobocyathus lagaensis* Barta-Calmus, 1969	middle Paleocene	Ivory Coast	Eastern Atlantic	[84]

^?^Questionable and unverifiable reports not included in Figs 8 and 9.

**Fig 7:**
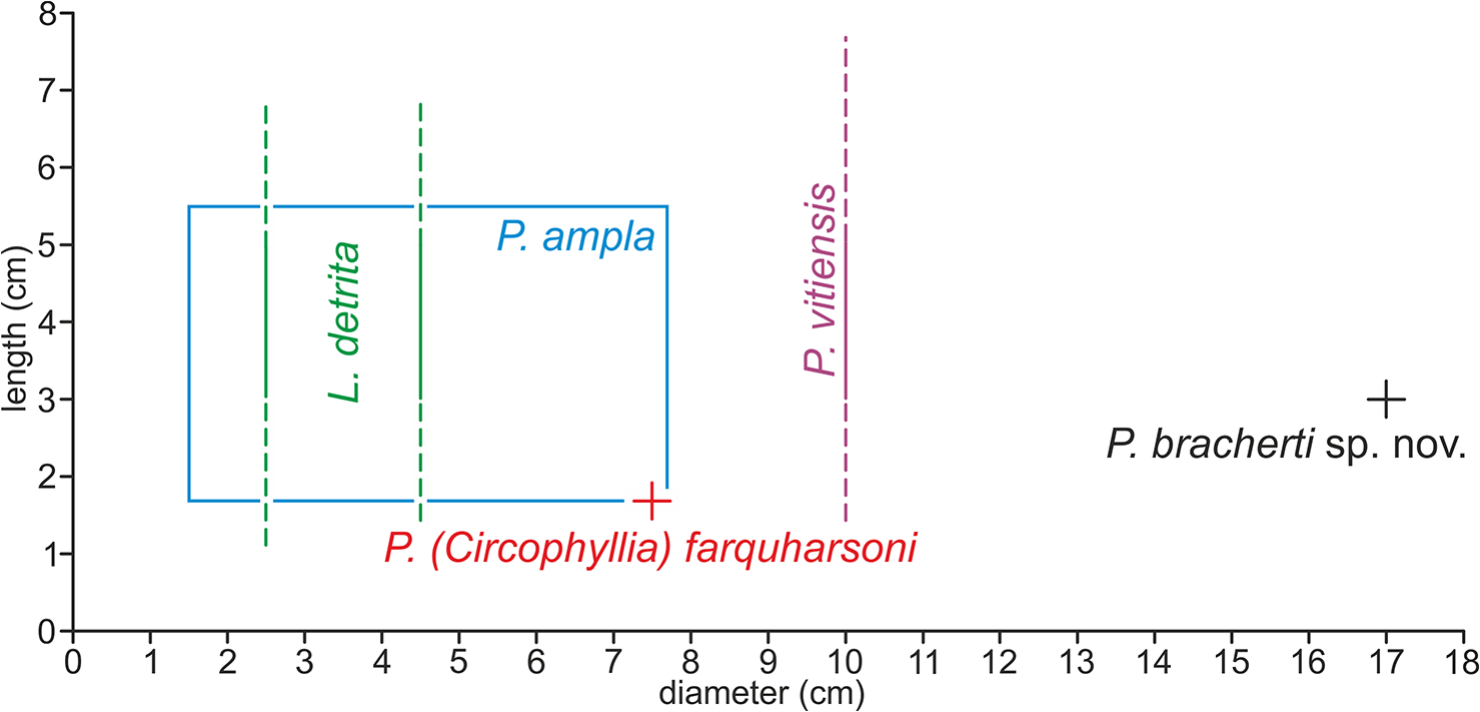
Size variation between different species of *Parascolymia* and *Lithophyllia detrita*. *Parascolymia vitiensis* (single calice specimens) [29,37], *Parascolymia ampla* (n = 13) [40,42,43,57], *Parascolymia (Circophyllia) farquharsoni* (n = 1) [51], *Lithophyllia detrita* (n = 11) [39]. The corallum of *P*. *vitiensis* is described without exact length measurements. The information that single calice specimens typically may reach 25 cm length has not been verified [36]. Similarly, the length of *Lithophyllia detrita* is not documented.

Although *Parascolymia bracherti* sp. nov. is only represented by one specimen, it can be clearly distinguished from the known *Parascolymia*, *Lithophyllia* and *Circophyllia* species by its significantly larger monocentric calice (170×170 mm; Fig 7). It should be stressed in that context that *Parascolymia* in recent high-latitude coral reef environments (comparable with the middle Miocene Central Paratethys) are smaller than their tropical counterparts [7,35,36]. The calice size is a significant criterion for the differentiation of fossil *Lithophyllia* species and recent Lobophylliidae genera [5,46,48]. The largest corallum of a *Parascolymia ampla*, which most closely resembles the new species, is that of the type specimen from the Badenian of Lăpugiu de Sus in Romania (77×67 mm in diameter; Fig 6). A collection of 10 further turbinate coralla from the same locality [43] documents lengths between 17‒45 mm and diameters of 15‒60 mm. Another poorly preserved discoid specimen from the Badenian of Hungary identified as cf. *Acanthophyllia ampla* has a calice diameter of 38×74 mm [42]. Furthermore, a poorly preserved cf. *Lithophyllia ampla* has been mentioned from the middle Miocene of Sarykawak in Turkey [57]. It has a calice diameter of 60×75 mm. The calice diameter of *Lithophyllia detrita* varies between 25 mm and 45×39 mm [39]. The type specimen of *Parascolymia (Circophyllia) farquharsoni* is 75×67 mm in diameter [54], while single calice specimens of the recent *Parascolymia vitiensis* can grow up to a diameter of 100 mm at tropical localities [7,35,36]. A further morphological feature that may distinguish *Parascolymia bracherti* sp. nov. from *Parascolymia ampla* is the ornamentation of the costa margin. The costae of *P*. *bracherti* sp. nov. show a blunt saw-like pattern (Fig 5C and 5D), while those of *P*. *ampla* have pronounced teeth (Fig 6B).

### Origin and Dispersal of *Parascolymia*–Understanding Cenozoic Trends of Marine Biodiversity

The palaeobiogeographic distribution of *Parascolymia* is generally obscured by the uncertain taxonomic status of potentially related *Circophyllia*, *Scolymia* and *Lithophyllia* fossils and their often poor stratigraphic constraints (Table 1). Important characters necessary for appropriate species delineation have not been preserved in several fossil species described from the Indo-Pacific region (*Lithophyllia spinosa*, *L*. *robusta*), which renders them questionable. Other dubious reports from this area (*C*. *d’archiardii*) cannot be checked for their reliability since they are solely based on fossil lists [54,55]. If, however, such questionable references will not be considered there is an obvious palaeobiogeographic pattern emerging, which has also been documented independently in other shallow marine organisms.

**Fig 8:**
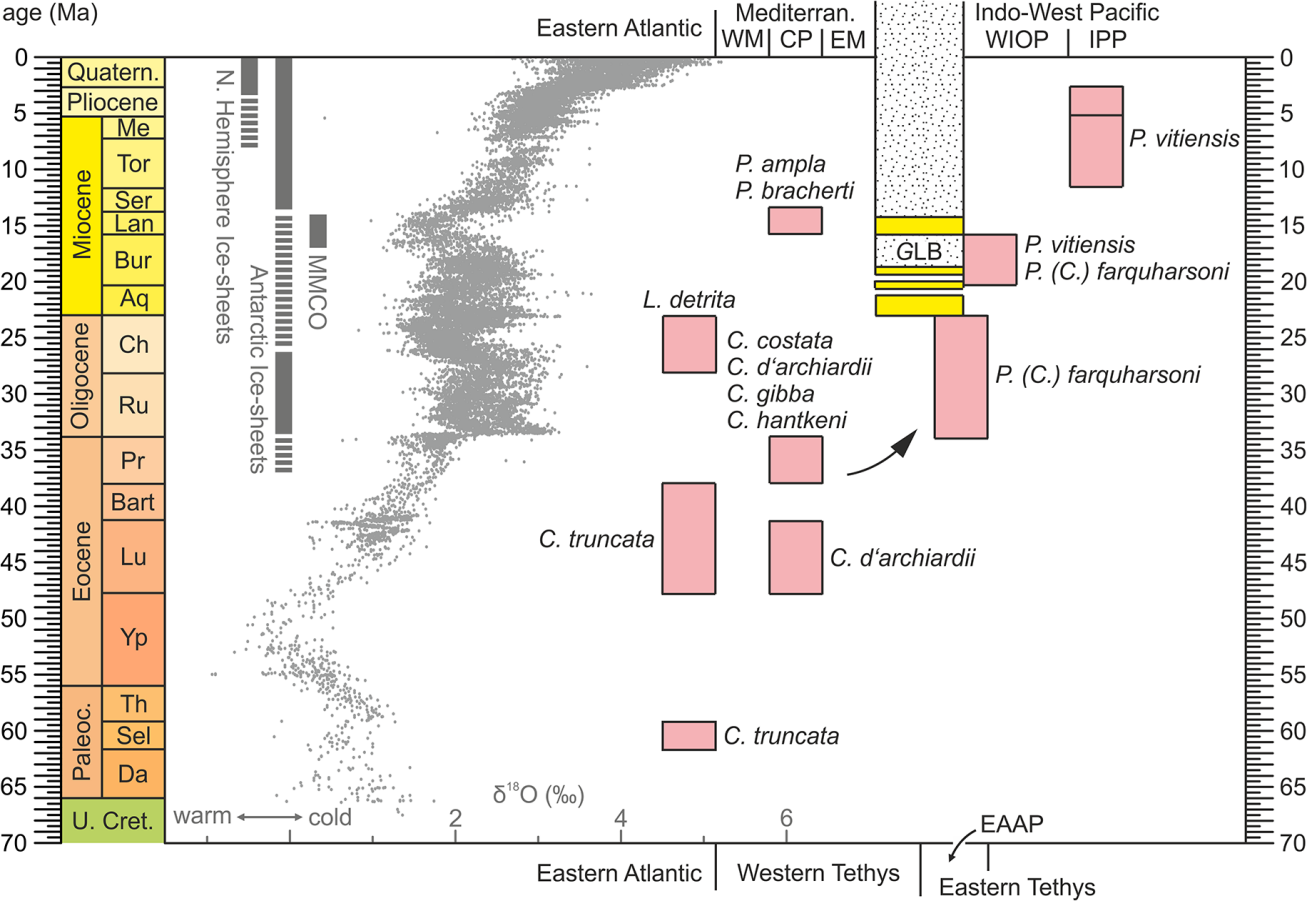
Correlation chart comparing the stratigraphic and biogeographic distribution of *Parascolymia* and possibly related coral taxa with the global climate trend, as reflected in the deep-sea oxygen isotope record [63,85], and with palaeogeographic events [13]. Coral occurrences refer to Table 1. The dotted pattern indicate a closed Tethyan Seaway and the intermittent yellow fields represent impaired marine connections impassable for shallow marine biota from the Eastern Tethys/Proto-Indo-West Pacific; EAAP = East African‒Arabian Province, WIOP = Western Indian Ocean Province, IPP = Indo-Polynesian Province, *G*LB = *Gomphotherium* Landbridge, MMCO = Middle Miocene Climate Optimum; chronostratigraphy according to [86].

The oldest reliable records of *Parascolymia* in the Indo-West Pacific region and the occurrences of *Parascolymia* in the Central Paratethys correlate with the time traditionally associated with the breakup of the Eocene pan-tropical Tethyan reef coral province into three provinces of the Western Atlantic‒Caribbean, Mediterranean and Indo-Pacific [14,58]. The Mediterranean to Western Indian Ocean region was a critical area for this biogeographic change through the collision of the Afro-Arabian plates with Eurasia interrupting the marine connection between the Eastern and the Western Tethys (Tethyan Seaway). The Mediterranean and Paratethys seas became practically isolated from the Indian Ocean since the late Burdigalian (ca. 19 Ma) due to the emergence of the “*Gomphotherium* Landbridge” connecting Africa with Eurasia (Fig 8) [13]. The expected divergence of shallow marine faunas (including reef corals) during the early Miocene is reflected on both sides of the seaway, but surprisingly the cut appears very early during the Aquitanian, i.e. 4‒5 Ma before the formation of the “*Gomphotherium* Landbridge” (Fig 8) [13,14,27,59]. Remarkably, shallow marine gastropod faunas from Oman, India and Tanzania also document a considerable provincialism within the western Indian Ocean area during the early Miocene and a surprisingly low relation to the Indonesian area [13,26,60,61]. This indicates a major biogeographic boundary between both regions. The occurrence of *Parascolymia* in the Central Paratethys Sea corresponds to a worldwide sea level highstand related to the Middle Miocene Climate Optimum [62,63], which re-opened the Tethyan Seaway for a short time (Fig 8) [16]. This short-lived marine connection via the Mesopotamian Trough provided potentially access for Indo-Pacific corals like *Parascolymia* into the Mediterranean‒Eastern Atlantic region. However, the biogeographic separation between Mediterranean and Indo-West Pacific shallow marine faunas (corals, molluscs) remained stable at that time [13,14,51,64]. Therefore, *Parascolymia bracherti* sp. nov. and *Parascolymia ampla* comb. nov. represent rather Mediterranean endemic species than immigrants from the Indian Ocean. This interpretation supports molecular genetic data suggesting that the Atlantic and Pacific clades of reef corals diverged long before the closure of the Tethyan Seaway [3]. In accordance with this finding, the fossil record of *Circophyllia* dates back into the middle Paleocene by the appearance of *C*. *truncata* [65] in West Africa and until the end of the Eocene Epoch the genus seems restricted to the Eastern Atlantic and Western Tethys region (Table 1, Fig 8).

**Fig 9:**
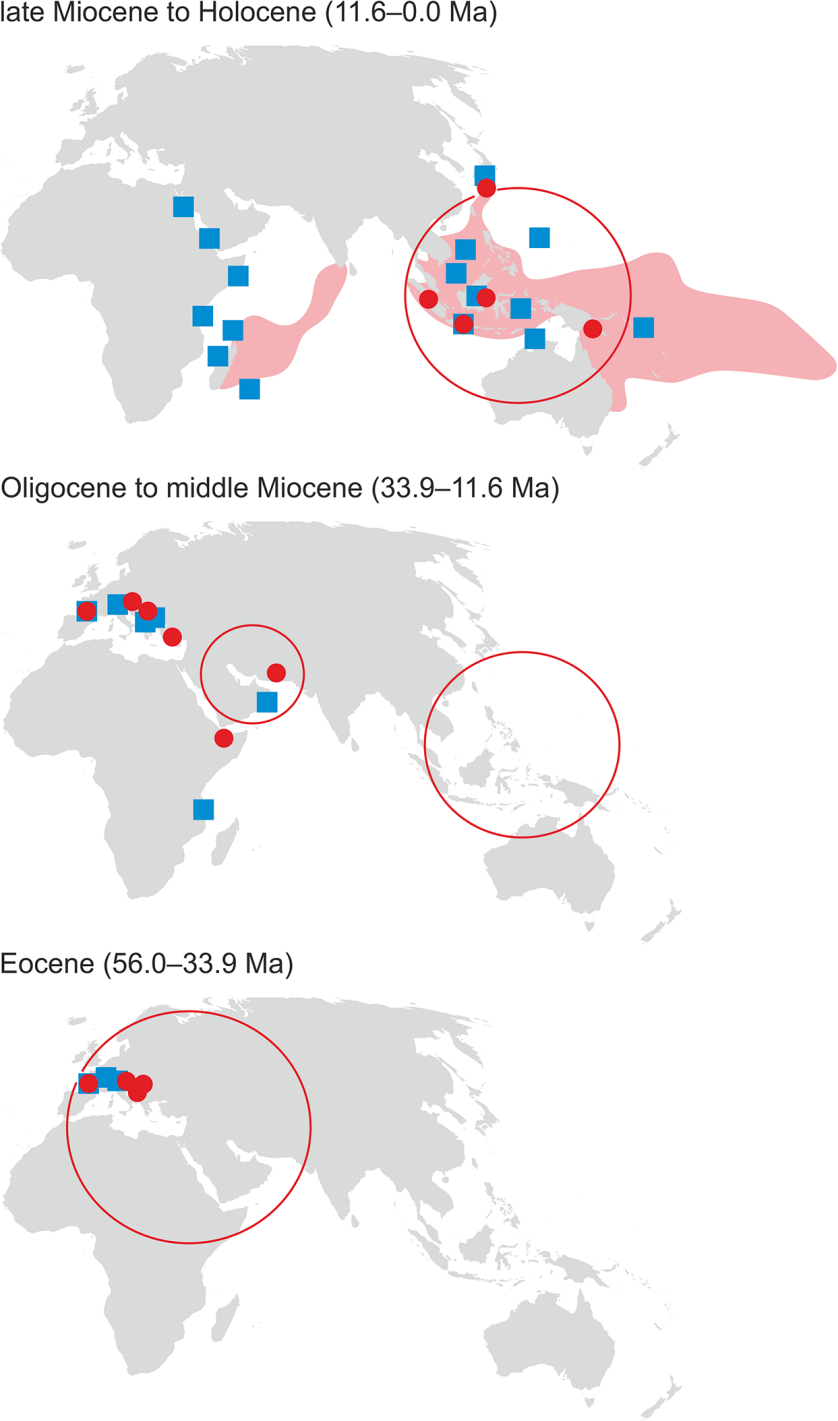
Geographic distributions of *Parascolymia/Circophyllia* (red dots) and tridacnines (blue squares,) in the Eocene, Oligocene to middle Miocene, and late Miocene to Holocene time slices. Coral occurrences refer to Table 1 and tridacnine distributions are compiled from [26,69]. The red shaded areas indicate the recent distribution of *Parascolymia vitiensis* [7] and the red circles delimit biodiversity hotspots in the respective periods [10].

The herein presented palaeobiogeographic pattern of *Parascolymia* shows obvious parallels with that of the reef coral *Acropora* and tridacnine bivalves (Fig 9). *Acropora* is presently found in all three major oceans of the world, with its center of diversity in the Indo-West Pacific. The fossil record, however, indicates that it originated and diversified in the North African‒Mediterranean area with oldest records from the late Paleocene of Italy, Austria and Somalia [66]. During the Eocene *Acropora* flourished in the Western Tethys and the Caribbean but was missing until the latest Oligocene to earliest Miocene in the Indo-West Pacific region [67,68]. Modern tridacnines appeared for the first time on the Arabian Shelf and in East Africa during the Chattian and Aquitanian, represented by *Omanidacna* and *Tridacna*, but their Eocene ancestors, which are related to *Byssocardium*, had an exclusively Western Tethyan distribution (Fig 9) [26,69]. From the contemporaneous occurrence of *Byssocardium* and *Omanidacna* during the late Oligocene it has been concluded that the separation of modern tridacnines from a *Byssocardium*-like ancestor may be rooted in the Eocene or early Oligocene [69]. Likewise, the occurrence of *Parascolymia (Circophyllia) farquharsoni* in the Oligocene of Somali and its association with *P*. *(Parascolymia) vitiensis* in the early Miocene of Makran [51,54] (Fig 8) suggest that the two subgenera diverged prior to the biogeographic isolation of the Mediterranean Sea from the Indian Ocean (Fig 6). Tridacnine bivalves [69] and probably also *P*. *(Parascolymia)* used the East African‒Arabian bioprovince as stepping stone into the Indo-Polynesian bioprovince and became typical elements of the entire Indo-West Pacific region after the closure of the Tethyan Seaway (Fig 8).

The spatio-temporal distributions of *Parascolymia*, *Acropora* and tridacnines strikingly reflect global movements in marine biodiversity through time (Fig 9). For the last 50 million years three geographically distinct hotspots of marine biodiversity have been identified [10]: (1) the Eocene West Tethyan hotspot, representing the place of diversification of *Parascolymia*; (2) the late Eocene‒early Miocene Arabian hotspot acting as stepping stone for *Parascolymia* into the Indo-West Pacific; and (3) the early Miocene‒recent Indo-Australian Archipelago hotspot in the center of the recent distribution of *Parascolymia* (Fig 9). This displacement of the marine center of biodiversity has been primarily attributed to major tectonic events controlling the availability, location and complexity of shallow water areas in the tropics [12, 10]. Some originally Western Tethys taxa such as *Parascolymia*, *Acropora* and tridacnines successfully managed to follow the moving center of marine biodiversity and formed the Miocene stock of the present Indo-West Pacific fauna. However, the gradual transition from the Western Tethys to the Indo-West Pacific reflects rather a continuous southwards shifting of the tropical center of maximum marine biodiversity in line with the Cenozoic climate deterioration (Fig 8) than a mainly tectonically-induced hopping [10]. This confirms the importance of long-term Cenozoic cooling for the decline of tropical shallow marine ecosystems in northern hemisphere mid-latitudes [14, 70] and hence for the shift of the marine biodiversity hotspot into the Middle East region by the late Eocene. De facto, only the final step of the long-range biodiversity displacement, which occurred across the Oligocene‒Miocene transition inside the Indo-West Pacific (Fig 9), might be basically linked to tectonic activities. These led to an extensive loss of vital shallow marine habitats along the Tethyan Seaway and the northwestern coast of India [71–73], but in turn created vast shallow water areas in Southeast Asia [12, 10].

## Conclusions

*Parascolymia bracherti* sp. nov. is described from the Leitha Limestone (Langhian‒early Serravallian) in the Vienna Basin (Central Paratethys, Austria) based on the mould of a single corallum. In order to overcome the mouldic preservation grey-scale inversion images and silicone rubber casts were applied for the identification of original macro- and micromorphological features (columella type, septocostae granulation, shape of septal dentation, costa margin ornamentation). The results show that coral moulds potentially prove the chance to study micromorphological characters of reef corals from shallow water limestones where skeletal aragonite is usually dissolved. This is important since the highest diversity of zooxanthellate corals occurs in pure carbonate shallow-water environments, but the fossil record of the Scleractinia is significantly biased in favour of well-preserved isolated corals from argillaceous lithologies representing unfavorable habitats of reduced coral diversity [74]. *Acanthophyllia ampla*, which occurred associated with the new species in the Central Paratethys, is placed in synonymy with *Parascolymia*. Before this evidence, *Parascolymia* was considered as strictly Indo-Pacific coral genus. The records from the Central Paratethys occur roughly 7 million years after the complete isolation of the Mediterranean from the Indian Ocean coral faunas by the restriction of the Tethyan Seaway. This finding and a review of the temporal and spatial distribution of other fossil corals related to *Parascolymia* indicate that the lineage originated in the Eastern Atlantic‒Western Tethys region during the Paleocene‒Eocene, corresponding to the former center of marine biodiversity. It also reveals an obvious temporal and spatial coincidence between the dispersal of *Parascolymia* and the displacement of the marine biodiversity hotspot into the present Indo-West Pacific region. *Parascolymia* is thus an example of a successful transformation of an originally Tethyan element contributing to the present biodiversity in the Indo-West Pacific. The gradual nature of this palaeobiogeographic change implies an important climatic control contrasting the hypothesis of a primarily tectonically driven hopping of geographically distinct biodiversity hotspots in the Cenozoic.
